# From Nail Biting to Osteomyelitis: A Pediatric Case of Distal Phalanx Infection Secondary to Onychophagia

**DOI:** 10.7759/cureus.109220

**Published:** 2026-05-19

**Authors:** Salah Riyadh, Ashlyn Dull, John Reynolds

**Affiliations:** 1 Emergency Department, Pikeville Medical Center, Pikeville, USA

**Keywords:** distal phalanx, onychophagia, osteomyelitis, paronychia, pediatric infection

## Abstract

Onychophagia, commonly referred to as nail biting, is a body-focused repetitive behavior that occurs most frequently in children and adolescents. Although often perceived as a harmless habit, repetitive trauma to the periungual tissues can compromise the integrity of the cutaneous barrier and increase susceptibility to secondary bacterial infection. Extension of infection into deep soft tissues with associated bone involvement is uncommon, particularly within the pediatric population.

We present the case of a 10-year-old female patient who arrived at the emergency department with progressive infection of the left third digit in the setting of chronic nail biting. The condition initially began as a hangnail that developed into paronychia and subsequently progressed to a felon despite outpatient treatment that included oral and topical antimicrobial therapy and incision and drainage. At the time of emergency evaluation, a substantial distal soft tissue defect with persistent drainage was observed. Plain radiographs demonstrated soft tissue disruption without clear evidence of early bony destruction. Subsequent magnetic resonance imaging identified a small distal fluid collection consistent with abscess formation and signal abnormalities of the distal phalanx suggestive of osteomyelitis. Given concern for deep infection, a hand surgery consultation was obtained, and the patient was transferred to a tertiary pediatric center. She was ultimately treated non-operatively with culture-directed antimicrobial therapy and close multidisciplinary follow-up, resulting in marked clinical improvement.

This case demonstrates a rare but significant complication of chronic nail biting in a pediatric patient and reinforces the need for careful assessment of worsening digital infections. Clinicians should consider advanced imaging and early specialty involvement when periungual infections do not respond to initial outpatient management, as prompt recognition and intervention are essential to minimize long-term morbidity and preserve function.

## Introduction

Onychophagia, or habitual nail biting, is a common body-focused repetitive behavior that begins most often in childhood and adolescence. Although frequently dismissed as a harmless habit, repeated mechanical trauma to the periungual region can compromise the protective nail fold and cuticle, creating a portal of entry for bacterial pathogens. Epidemiologic studies estimate that nail biting affects approximately 20-30% of school-aged children and adolescents, with prevalence declining into adulthood [1].

Disruption of the periungual barrier predisposes patients to acute paronychia, an infection involving the nail fold and adjacent periungual tissues. Extension of infection into the compartmentalized distal pulp space may result in a felon, a deeper fingertip infection characterized by pain and swelling [2]. While these infections are typically superficial and responsive to conservative treatment, ongoing manipulation or delayed recognition may permit extension into deeper soft tissues. In rare cases, infection may involve the distal phalanx, resulting in osteomyelitis, a complication that has been described in isolated reports associated with nail biting behaviors [3].

Given the potential for progression despite initially mild presentation, awareness of this complication is particularly important in pediatric patients with worsening or refractory digital infections. The following case illustrates the development of distal phalanx osteomyelitis in a child with chronic onychophagia and highlights key considerations in early recognition and management.

## Case presentation

A 10-year-old female patient presented to the emergency department at Pikeville Medical Center with worsening pain, drainage, and tissue breakdown involving the left third digit. Her medical history was notable for chronic nail biting. Approximately 10 days prior to presentation, she developed a hangnail on the affected finger that became progressively erythematous and tender. She was evaluated by her pediatrician and diagnosed with acute paronychia, for which oral amoxicillin-clavulanate was prescribed.

Two days later, due to increasing pain and swelling, she returned for reevaluation and was diagnosed with a felon. Incision and drainage were performed, and antimicrobial therapy was broadened to include trimethoprim-sulfamethoxazole and topical mupirocin. Despite these interventions, she experienced persistent drainage and progressive distal tissue deterioration over the following five days, prompting referral to the emergency department for further assessment.

**Figure 1:**
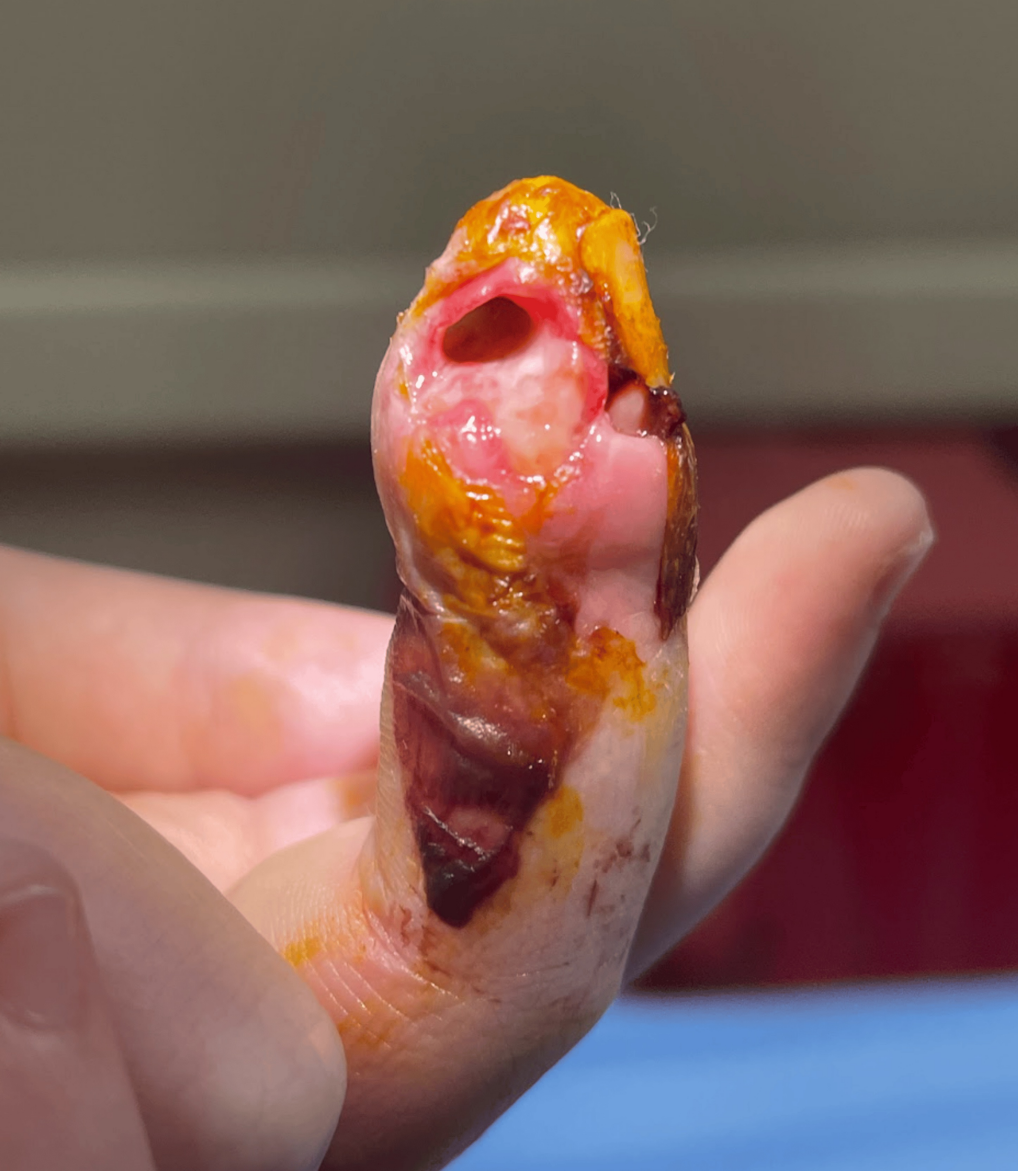
Medial aspect of the left third digit at emergency department presentation Clinical photograph demonstrating a distal soft tissue defect of the medial aspect of the left third digit with exposed underlying tissue, surrounding erythema, and areas of tissue discoloration concerning for severe infection. Active drainage was present at the time of evaluation.

**Figure 2:**
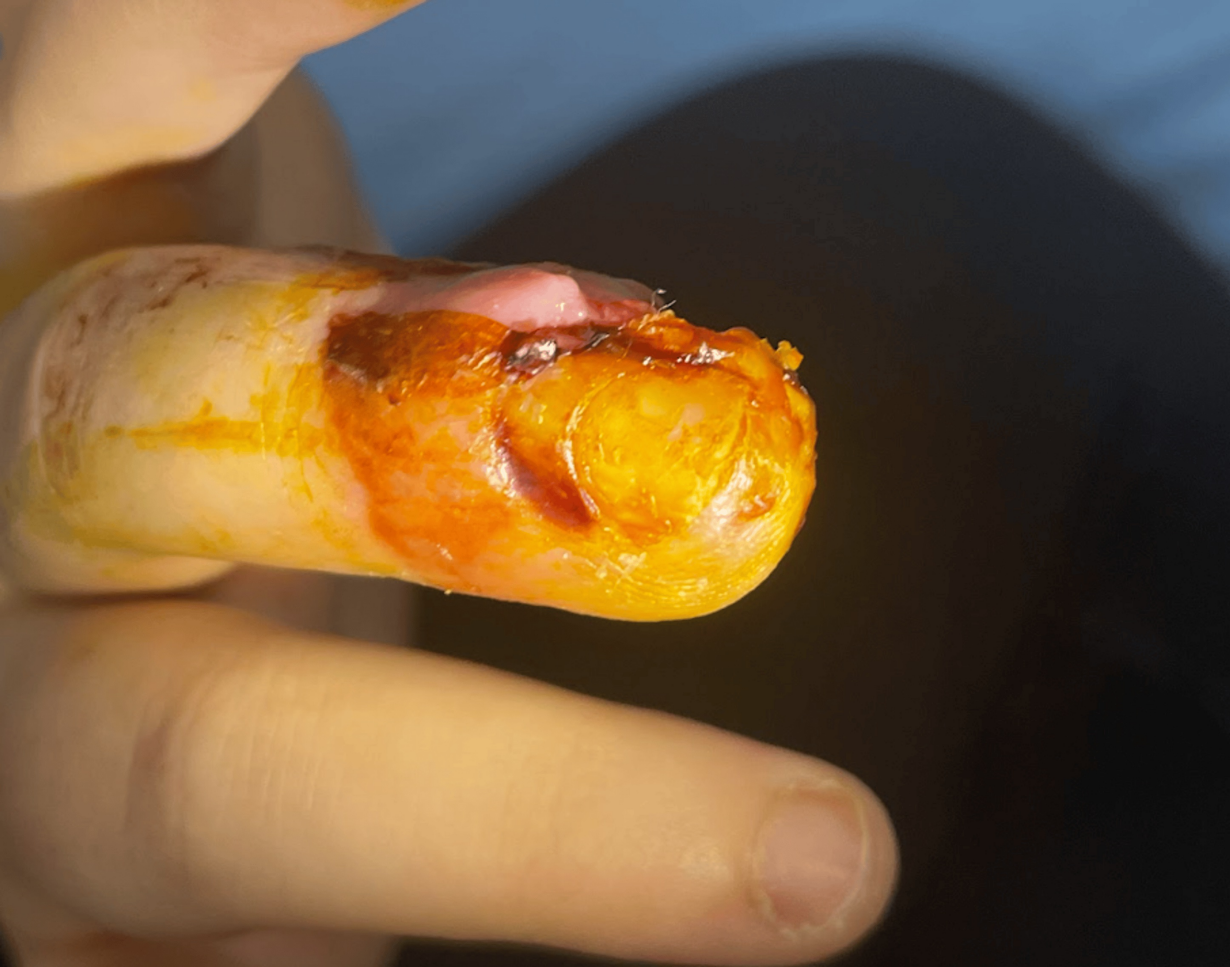
Posterior aspect of the left third digit demonstrating soft tissue injury Clinical photograph showing the posterior aspect of the left third digit with significant soft tissue disruption, surrounding erythema, and tissue discoloration at the fingertip following prior incision and drainage. The image highlights the extent of distal tissue involvement at the time of emergency department evaluation.

At presentation, she denied fever, chills, nausea, vomiting, or other systemic symptoms. Vital signs were within normal limits for age. Physical examination revealed an approximately 1.5 cm distal soft tissue defect (Figures 1-2) involving the left third digit with exposed underlying tissue, active purulent drainage, surrounding erythema, and areas of tissue discoloration. Capillary refill was intact, sensation was preserved, and no gross neurovascular compromise was identified.

Laboratory studies, including complete blood count and inflammatory markers, were not obtained during the initial emergency department evaluation. The patient was clinically stable and expressed significant distress regarding phlebotomy, and transfer to a tertiary pediatric center for further management had already been arranged.

**Figure 3:**
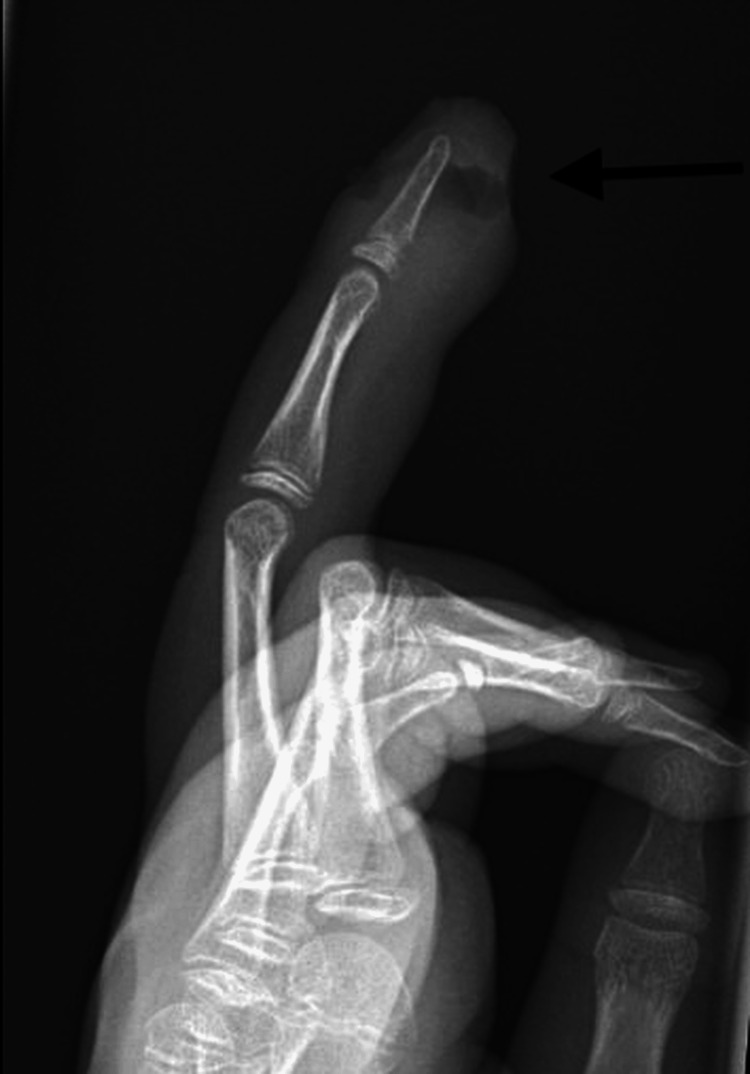
Lateral radiograph of the left third digit demonstrating distal soft tissue defect Lateral plain radiograph of the left hand demonstrating a soft tissue defect involving the distal aspect of the left third digit (arrow). No acute fracture or dislocation is identified on this view.

Plain radiographs of the left hand (Figure 3) demonstrated a distal soft tissue defect with associated swelling of the third digit. No acute fracture, dislocation, or definitive early osseous destruction was identified on initial imaging.

Given the limited sensitivity of plain radiography in early osteomyelitis, underlying bone involvement could not be excluded. Wound cultures obtained from prior drainage at the pediatrician's office grew methicillin-resistant *Staphylococcus aureus*, which was susceptible to clindamycin, trimethoprim-sulfamethoxazole, tetracycline, and vancomycin.

Due to concern for deep soft tissue infection with possible osseous extension, hand surgery was consulted, and the patient was transferred to a tertiary pediatric center for advanced evaluation. Magnetic resonance imaging of the left hand demonstrated a distal soft tissue defect with surrounding edema consistent with cellulitis, a small volar fluid collection suggestive of abscess formation, and signal abnormalities within the distal phalanx concerning for acute osteomyelitis.

She was initiated on intravenous vancomycin and piperacillin-tazobactam and evaluated by orthopedic hand surgery and pediatric infectious disease specialists, who confirmed the diagnosis of acute osteomyelitis. Operative intervention was deferred in favor of medical management. Based on culture susceptibilities, she was transitioned to oral doxycycline and completed a three-week course of antimicrobial therapy.

At the three-week outpatient infectious disease follow-up, she reported complete resolution of pain and drainage with preserved digital function. Mild residual scarring was noted, with anticipated gradual improvement. Repeat radiographs demonstrated persistent soft tissue swelling and osteolysis of the distal phalanx consistent with resolving osteomyelitis, as well as changes of the middle phalanx suggestive of disuse osteoporosis. No findings indicated ongoing active infection, and additional antimicrobial therapy was not recommended. Further infectious disease follow-up was deemed unnecessary.

## Discussion

Onychophagia is commonly perceived as a benign behavioral habit; however, repeated trauma to the periungual region can compromise the integrity of the nail fold and cuticle, facilitating bacterial entry into deeper tissues [4]. Barrier disruption predisposes patients to acute paronychia and, when infection extends into the distal pulp space, felon formation [2]. Although most periungual infections remain localized, persistent manipulation or delayed treatment may allow progression beyond superficial structures. In rare instances, infection may involve the distal phalanx, leading to osteomyelitis, a complication that has been described in isolated reports associated with nail biting behaviors [3]. The present case is notable because of the uncommon progression from chronic nail biting to distal phalanx osteomyelitis in a pediatric patient despite an initially mild presentation and absence of systemic symptoms.

Digital infections related to nail trauma are frequently caused by *Staphylococcus aureus*, though behaviors such as nail biting may also introduce oral flora into periungual tissues [4]. In pediatric patients, the absence of systemic symptoms can contribute to delayed recognition of deeper infection. Progressive tissue destruction, ongoing drainage, or failure to respond to appropriate outpatient therapy should prompt further diagnostic evaluation.

Radiographic imaging is often obtained early in the evaluation of suspected digital infection. However, plain radiographs may be normal during the initial stages of osteomyelitis [5]. When clinical suspicion remains high despite nondiagnostic radiographs, magnetic resonance imaging is considered the most sensitive modality for detecting early bone marrow changes and associated soft tissue involvement [5]. In the present case, advanced imaging confirmed distal phalanx involvement and guided management decisions.

Management of complicated digital infections typically involves collaboration among emergency clinicians, surgical specialists, and infectious disease teams. Early surgical consultation should be considered when there is concern for deep space infection, tissue necrosis, or possible osseous extension. In carefully selected pediatric patients who remain clinically stable, non-operative management with culture-directed antimicrobial therapy and close follow-up may be appropriate.

Behavioral and psychiatric assessment may be considered in selected patients with severe, recurrent, or functionally impairing body-focused repetitive behaviors. However, a formal psychological evaluation was not performed in the present case because the patient was evaluated in an acute emergency setting and transferred for definitive care. 

Addressing contributory behavioral habits is an important component of comprehensive management and may reduce the risk of recurrence. Behavioral interventions such as habit reversal training have demonstrated benefit in some patients with onychophagia and may represent an important component of long-term management [4]. Pharmacologic interventions, including selective serotonin reuptake inhibitors, have been described in selected patients with body-focused repetitive behaviors; however, management is individualized and generally guided by formal psychiatric evaluation.

## Conclusions

This case illustrates an uncommon but clinically meaningful complication of chronic nail biting in a pediatric patient. Although onychophagia is often perceived as benign, repeated disruption of the periungual barrier may permit progression from superficial infection to deeper soft tissue and osseous involvement. In this patient, worsening symptoms despite outpatient management ultimately revealed distal phalanx osteomyelitis confirmed by advanced imaging.

Clinicians should remain attentive to persistent or progressive periungual infections in children, even in the absence of systemic illness. Consideration of deeper infection, appropriate imaging when clinically indicated, and timely specialty involvement may help reduce the risk of long-term morbidity. Addressing contributory behavioral habits is an important component of comprehensive management and prevention of recurrence.
